# Nursing care of a patient with abdominal wall abscess caused by *Granulicatella adiacens* infection due to improper insulin injection: A case report

**DOI:** 10.1097/MD.0000000000042408

**Published:** 2025-05-09

**Authors:** Jun Wang, Wei Wei, Yao Peng, Jia Li, Huan Li

**Affiliations:** a Center for Infectious Diseases, West China Hospital, Sichuan University/West China School of Nursing, Sichuan University, Chengdu, China.

**Keywords:** abdominal wall abscess, *Granulicatella adiacens*, nursing, wound healing

## Abstract

**Rationale:**

*Granulicatella adiacens* (*G adiacens*) are colonizing bacteria commonly found in the mouth, intestine, and urogenital tract. These bacteria tend to cause infectious diseases in immunocompromised hosts, such as artificial joint infections, osteomyelitis, meningitis, peritonitis, and lung abscess.

**Patient concerns and diagnoses:**

An unusual case involved a 37-year-old female who developed an abdominal wall infection adjacent to *G adiacens* due to incorrect insulin injection behavior.

**Interventions:**

The patient received targeted anti-infection treatment, including wound dressing changes and blood glucose management.

**Outcomes:**

Her symptoms, which included fever and a skin rupture with pus and bleeding on the left lower abdomen, improved markedly. She was discharged after 14 days of hospitalization, and her abdominal wound fully healed 5 months later.

**Lessons:**

This case underscores the importance of prompt identification and careful wound care. The nurse practitioner played a critical role in specimen collection, wound management, antibiotic administration, and patient education. Integrated care and interdisciplinary collaboration were key factors that led to the patient’s significant improvement and successful discharge.

## 1. Introduction

The diabetes has become a significant public health issue, and the global burden of disease study shows that there are 529 million diabetic patients in the world in 2021, and the number is expected to rise to 1.31 billion by 2050.^[1]^ The estimated number of individuals affected by diabetes in China reached 140.8 million in 2021, with projections from the International Diabetes Federation indicating an increase to 174.4 million by 2045.^[2]^ Long-term insulin therapy is essential in patients with diabetes.

Abdominal wall abscess is a rare local complication of subcutaneous insulin application. *Granulicatella adiacens* (*G adiacens*) are tachylase-negative, oxidase-negative, and facultatively anaerobic gram-positive bacteria that belong to the genus *Granulicatella*. Also known as nutritional variant *Streptococcus*, this demanding pathogen requires vitamin B6 analogues and l-cysteine for growth.^[3]^ These bacteria are commonly found in the mouth, intestine, and urogenital tract.^[4]^ *G adiacens* can easily cause disease and frequently leads to bloodstream infections and infective endocarditis in immunocompromised hosts. It has typically been reported as a cause of bacteremia and endocarditis in adults with comorbidities.^[5]^ Infections caused by *G adiacens* have predominantly been documented as case reports, including infections of prosthetic valves,^[6]^ pacemaker electrodes,^[7]^ artificial joints,^[8,9]^ peritonitis,^[10]^ lung abscess,^[11]^ osteomyelitis,^[12]^ and spinal osteomyelitis.^[13]^ However, there are no reported cases of abdominal wall abscesses caused by *G adiacens*. Additionally, previous case reports have focused primarily on patient treatment, with limited discussion on the key aspects of nursing care. This report presents a rare case of a 37-year-old female patient with abdominal wall infection adjacent to *G adiacens* due to incorrect insulin injection behavior, and demonstrated the nursing experience during the treatment.

## 2. Case report

On March 6, 2023, a 37-year-old female patient was admitted to the hospital with complaints of left lower abdominal wall swelling, fever persisting for 8 days, and an abscess requiring incision and drainage for 3 days. Eight days prior to admission, she experienced redness, swelling, and pain at the site of a nonstandard subcutaneous insulin injection. Issues included failure to change the insulin needle, inadequate disinfection of the injection site, and irregular site rotation. She also had a fever with a maximum temperature of 39 °C. Despite oral amoxicillin treatment, her symptoms did not improve, which prompted her to seek care at a local hospital. A computerized tomography scan of the abdominal wall revealed infection of the left lower middle abdominal wall and the potential of abscess formation. An incision was made, and approximately 400 mL of yellow–brown pus was drained. However, the patient’s local symptoms persisted, and pain remained significant. She was subsequently transferred to our department 3 days after the initial incision for further evaluation and treatment.

## 3. Diagnosis

**Figure 1.**
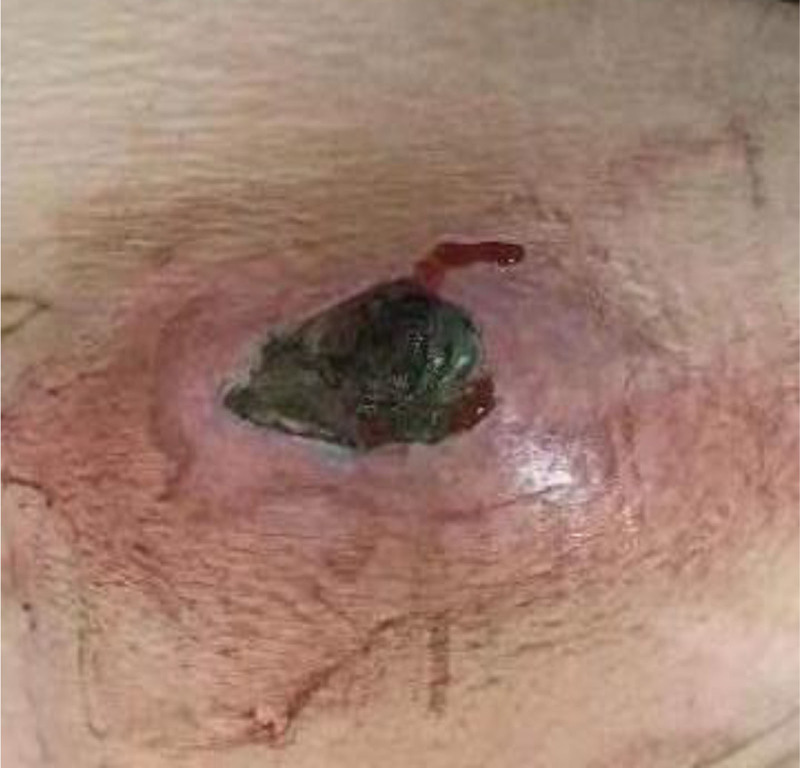
Incision in the abdominal wall after admission.

Upon admission, vital signs were recorded: body temperature 37.1 °C, pulse 92 beats/min, respiratory rate 20 breaths/min, blood pressure 131/88 mm Hg, height 155 cm, weight 81 kg, body mass index 33.71 kg/m² and random blood glucose level 15.8 mmol/L. Physical examination revealed the incision site on the abdominal wall covered with black–brown necrotic tissue, characterized by erythema, exudate, and a foul odor. The local skin was markedly disrupted, with surrounding skin exhibiting a purple–red discoloration (Fig. 1). Blood tests showed a C-reactive protein level of 102 mg/L (normal reference value: <5 mg/L), interleukin-6 level of 29.6 pg/mL (normal reference value: 0–7 pg/mL), procalcitonin level of 0.23 ng/mL (normal reference value: <0.046 ng/mL), glycated hemoglobin A1c level of 12.7% (normal reference value: 4.5%–6.1%), and albumin level of 30.3 g/L (normal reference value: 40–55 g/L).

## 4. Interventions

On the 2nd day after admission, the patient presented significant swelling in the left lower abdominal wall. Upon comprehensive assessment, further incision was deemed unnecessary. Instead, surgical wound cleaning and dressing change were sufficient to facilitate pus drainage with collaboration between doctor and nurse. Prior to this procedure, an intramuscular injection of 5 mg dezocine was administered for analgesia due to the patient’s pain level of 4 on the numerical rating scale (NRS), given the ineffectiveness of local anesthesia for pain control in infected wounds. Thirty-minute postinjection, approximately 60 mL of black–brown blood clots were expelled from the abscess incision as the doctor applied pressure around the wound. Subsequently, about 400 mL of red and white pus was drained as well. Samples for microbiological culture were sent to the laboratory. Due to the substantial exudate from the patient’s abscess, the dressing adhered to the wound. The nurse moistened the entire layer of the dressing with 0.9% normal saline before gently removing it. A 20 mL syringe was used to draw up physiological saline for gentle irrigation of the wound, then the black necrotic tissue was removed with sterile scissors. The black–brown eschar and residual pus were excised, and the wound was covered with sterile gauze soaked in a 1:10 dilution of povidone–iodine (0.5% povidone–iodine). Cotton pads were placed over the gauze. After comprehensive cleaning and disinfection, 20 mL of pus was aspirated and cultured for both aerobic and anaerobic organisms. Additionally, intravenous imipenem/cilastatin 1 g every 8 hours was administered. Before administration, the nurse informed the patient about the drug’s name, effects, purpose, and the necessary cooperation, to ensure that the patient and her family were fully briefed. The nurse scheduled and administered the infusion on time to maintain effective blood concentration. During intravenous administration, the nurse closely monitored the patient for severe adverse reactions, such as rash, dyspnea, or vomiting. Any adverse reactions were promptly reported to the doctor for timely intervention.

On the 4th day after admission, fasting blood glucose and postprandial blood glucose levels were monitored, measuring 17.2 mmol/L and 20.9 mmol/L, respectively. Following consultation with an endocrinologist, the treatment regimen included subcutaneous injections of 10 units of insulin aspart before each meal, 12 units of insulin glargine before bedtime, and 1.2 mg of liraglutide daily. Before administration of these injections, the nurse provided detailed instructions about drug precautions. For example, insulin aspart should be injected shortly before meals, with food consumed within 15 minutes to prevent hypoglycemia. The nurse also educated the patient about the symptoms of hypoglycemia to ensure she could seek medical assistance if needed. Additionally, given that the disease stemmed from improper insulin injection practices, the nurse explained the correct techniques for insulin administration, including proper site selection and rotation, site disinfection, and the use of a new needle for each injection.

When administering liraglutide subcutaneously, the nurse informed the patient about potential adverse reactions, including abdominal distension, nausea, and vomiting. The patient indeed developed symptoms of upper abdominal distension, which the nurse closely monitored and managed. After 5 days of small-dose injections, these symptoms were relieved. Gastrointestinal adverse reactions associated with liraglutide are common and may include nausea, diarrhea, vomiting, constipation, and abdominal pain.^[14]^ In this case, the patient reported abdominal pain on the 2nd day of liraglutide use. A painless gastroscopy was promptly arranged by the physician, and revealed chronic non-atrophic gastritis, which was likely related to the pain and satiety induced by liraglutide.

In addition to monitoring for adverse drug reactions (e.g., from insulin and liraglutide), the nurse also tracked fasting blood glucose levels and postprandial blood glucose levels 2 hours after each meal to assess the effectiveness of the medication and detect and manage serious issues such as hypoglycemia. Table 1 displays the fasting plasma glucose and 2-hour postprandial glycemia levels during hospitalization.

**Table 1:** Fasting plasma glucose and 2-hour postprandial glycemia during hospitalization.

Time	FPG	2 h PPG (breakfast)	2 h PPG (lunch)	2 h PPG (dinner)
Day 2	14.3	15.5	21	19.4
Day 3	13.4	16.7	15.7	12.9
Day 4	17.2	18.7	20.9	14.7
Day 5	14.5	13.8	8.8	13.2
Day 6	11.7	18.2	16.2	15.4
Day 7	11.7	17.4	14.8	15.4
Day 8	13	18.5	17.3	16.4
Day 9	11.7	17.9	15.2	13.3
Day 10	9.5	16.1	11.7	10.4
Day 11	10.4	11.8	12.9	10.4
Day 12	10	16	11.2	13.3
Day 13	10.3	17.7	11.1	11.9
Day 14	7.1	17.5	11.6	9.9

FPG = fasting plasma glucose, 2 h PPG = 2-hour postprandial glycemia.

On the 7th day after admission, *G adiacens* was identified from the pus culture, and the antibiotic susceptibility test indicated sensitivity to levofloxacin. Consequently, imipenem/cilastatin was de-escalated to levofloxacin, administered intravenously at 0.1 g daily.

**Figure 2.**
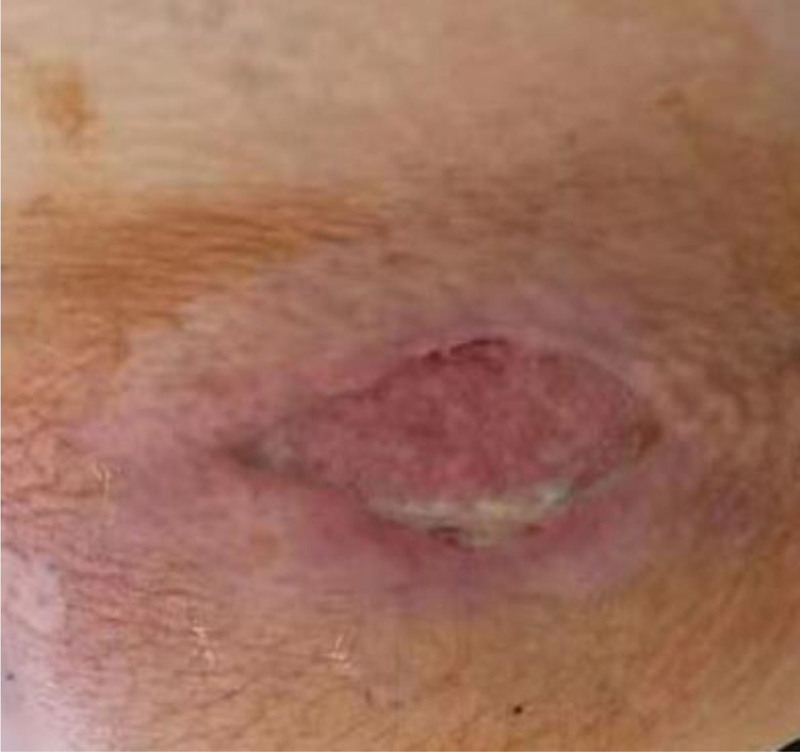
Incision on the abdominal wall on the 8th day after admission.

On the 9th day after admission, the wound showed significant improvement due to effective wound dressing, blood glucose control, and anti-infective therapy. The necrotic tissue was nearly removed, and fresh granulation tissue had developed at the base of the lesion (Fig. 2). The patient did not experience any fever.

On the 11th day after admission, the abdominal wall wound had recovered well, with no exudate or pain reported. Blood tests revealed a white blood cell count of 5.66 × 10^9^/L, C-reactive protein of 4.67 mg/L, and procalcitonin of 0.05 ng/mL. Therefore, the treatment was adjusted to oral levofloxacin 0.5 g daily.

On the 13th day after admission, the nurse provided health education to the patient for home care, assisting her in mastering insulin injection techniques, the selection of injection sites, and the rotation plan for these sites. Particular emphasis was placed on correcting previous improper practices, such as the reuse of insulin needles and inadequate disinfection of injection sites. Subsequently, the patient was required to self-administer insulin injections while the nurse observed her technique to ensure proper execution. Additionally, the patient was guided on weight management. The importance of controlling carbohydrate intake was emphasized, and the patient was encouraged to select low-glycemic-index carbohydrate sources, such as whole grains and legumes. The patient was also encouraged to engage in moderate-intensity aerobic exercises, such as brisk walking or jogging, at least 3 times per week, with each session lasting no <20 minutes. Blood glucose monitoring before and after exercise was recommended to ensure safety and optimize the benefits of physical activity.

## 5. Outcomes

**Figure 3.**
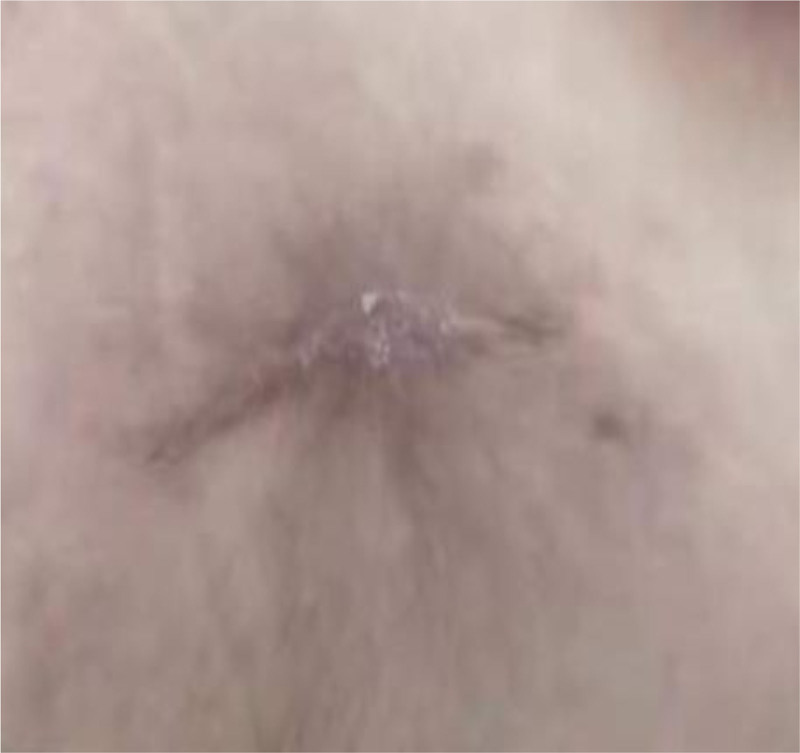
Incisional wound in the abdominal wall 5 months after discharge.

The patient was discharged on the 14th day after admission. She was advised to continue taking oral levofloxacin and to return for follow-up in 2 weeks. Additionally, she was instructed to monitor and control her blood glucose levels with insulin, consult endocrinology for regular insulin dose adjustments, and visit the local hospital for wound dressing changes. At the 5-month follow-up at the outpatient clinic, the wound had completely healed (Fig. 3).

## 6. Discussion

Although abdominal wall abscesses can occasionally develop along the transabdominal incision after open surgery, they are rarely caused by other conditions. However, other possible causes of abdominal wall abscesses include malignant tumors, acute appendicitis, gynecological disorders, Crohn disease, colonic diverticula, cholecystitis, and intestinal perforation.^[15]^

Solitary abdominal wall abscesses that are not secondary infections—referred to as primary abdominal wall abscesses (PAWAs)—are extremely rare. The mechanism underlying PAWAs remains unclear. Previous reports have identified muscle damage, diabetes mellitus, and liver cirrhosis as potential risk factors for PAWAs.^[16]^ To date, the understanding of infections caused by *G adiacens* has been limited, with abscesses typically reported as case studies (e.g., lung abscesses, parapharyngeal abscess).^[5,11]^

The treatment of abdominal wall abscesses typically involves surgical drainage and antibiotic therapy. This patient underwent incision and drainage of the abscess at an external facility. However, the wound healed poorly, necessitating appropriate dressing changes to facilitate faster healing. Current literature includes limited studies on the use of wound dressings specifically for skin and soft tissue infections caused by *G adiacens*. Regarding wound dressing options, some studies have demonstrated that silver nanoparticles (AgNPs) exhibit significant antibacterial activity against both Gram-positive and Gram-negative bacteria and inhibit biofilm formation.^[17]^ The antimicrobial properties of AgNPs have been confirmed in various in vitro and in vivo studies, and their applications extend to food packaging, soaps, cosmetics, textiles, and wound dressings.^[18]^ Consequently, silver is often considered a first-line option for topical wound infection treatment.^[18]^ However, due to the high cost of silver ion dressings and the financial constraints faced by the patient, a more affordable alternative was chosen: gauze dressings with polyvinylpyrrolidone iodine. Compared to silver, povidone–iodine has a broader spectrum of antimicrobial activity, effective biofilm control, and no known bacterial resistance or cross-resistance. It also has low cytotoxicity, good tolerability, and promotes wound healing, making it a viable option for wound care and biofilm management.^[19]^ Thus, povidone–iodine soaked gauze was applied to the patient’s wound, with a foam dressing used as a secondary layer to absorb wound exudate, maintain a moist environment, and promote the growth of granulation tissue. Currently, AgNPs are prioritized for wound infection control. However, the results in this case suggested that using povidone–iodine soaked gauze combined with a foam dressing might be a promising, cost-effective alternative. During wound dressing changes, the nurse also addressed the issue of operative pain, defined as the unpleasant pain experienced during dressing changes, including the removal or application of dressings, debridement, diagnosis, treatment, or nursing procedures.^[20]^ Frequent and prolonged dressing changes, coupled with inadequate pain management, can cause significant psychological stress and anxiety for patients, reduce their quality of life, and delay wound healing.^[21]^ To mitigate this, the nurse assessed pain using the NRS before and after dressing changes and administered effective analgesia, such as medication, based on the assessment results. This approach positively impacted the patient’s comfort and compliance with the dressing changes. As the wound improved, the patient’s pain significantly decreased, from a pain level of 4 on the NRS at admission to no pain at discharge.

Additionally, the patient’s skin became thinner and had impaired healing ability due to her type 2 diabetes. Trauma to such skin could lead to refractory wounds, chronic ulcers, potential amputation, and life-threatening conditions. Therefore, delayed wound healing is a common complication of diabetes.^[22]^ Glycemic control is crucial for wound healing. It has been well established that combining insulin with liraglutide effectively controls blood glucose and reduces body weight in patients with poor glycemic control despite high doses of insulin, or those with normal fasting glucose but high postprandial glucose and HbA1c levels.^[23,24]^

Previous studies have shown that demonstrating proper insulin injection techniques improves adherence in people with type 2 diabetes.^[25]^ In this case, incorrect injection practices—such as inadequate disinfection of the injection site and reuse of needles—contributed to the development of the disease. Therefore, instruction on standard insulin injection techniques was a crucial component of health education, helping to prevent recurrence of abdominal wall abscesses due to improper injection practices. After discharge, the patient did not experience any further abdominal wall infections.

In the treatment of abdominal abscesses, systemic anti-infective therapy is crucial, including early identification of pathogens and the use of antibiotics to which the bacteria are sensitive, in addition to incision and drainage. This requires standardized specimen collection and laboratory testing techniques. As the executor of the antibiotic protocol, the nurse’s responsibilities include evaluating potential drug allergies, adhering to the physician’s orders, adjusting infusion rates as needed, monitoring drug efficacy and side effects, regularly checking liver and kidney function, and communicating observation results to the physician. Additionally, the nurse should aim to minimize collateral damage from antibiotics, such as superinfections like pseudomembranous enteritis. After discharge, the patient continued oral antibiotics (levofloxacin). Research showed that a significant proportion of antibacterial agents were used by patients outside hospital settings, with common irrational practices including self-medication, improper storage, dose adjustments without medical advice, and discontinuation of medication.^[26–28]^ In this case, the patient initially took amoxicillin. Thus, providing the patient with knowledge about the rational use of antibacterial drugs was crucial to ensure adherence to treatment, particularly with oral levofloxacin during and after hospitalization. The patient experienced no antibiotic-related adverse reactions during hospitalization and adhered to the oral antibiotic regimen after discharge, indicating that the anti-infection plan was effective.

## 7. Conclusions

Abdominal wall abscesses caused by *G adiacens* are infrequently encountered, which underscores the importance of prompt identification of the causative microorganism and the implementation of judicious wound care in optimizing patient outcomes. Nursing professionals played a key role in collecting microbial specimens, selecting appropriate wound dressings, rational use of antibiotics, monitoring blood glucose levels, and health education to patients. Through an integrated approach encompassing comprehensive care and effective interdisciplinary collaboration, the patient experienced a marked improvement in her condition, culminating in a successful discharge from the healthcare facility.

## Author contributions

**Conceptualization:** Jun Wang, Wei Wei, Huan Li.

**Project administration:** Yao Peng, Jia Li.

**Supervision:** Wei Wei, Huan Li.

**Writing – original draft:** Jun Wang.

**Writing – review & editing:** Wei Wei, Huan Li.
